# Fatherhood among healthcare workers: perceived work–family conflict and its influential factors during the COVID-19 pandemic: cross-sectional and exploratory longitudinal findings from the VOICE study

**DOI:** 10.3389/fpubh.2026.1782679

**Published:** 2026-06-08

**Authors:** Sabine Mogwitz, Jona Körber, Lucia Jerg-Bretzke, Christian Albus, Andreas M. Baranowski, Petra Beschoner, Yesim Erim, Franziska Geiser, Eva Morawa, Susann Steudte-Schmiedgen, Gloria-Beatrice Wintermann, Kerstin Weidner

**Affiliations:** 1 Department of Psychotherapy and Psychosomatic Medicine, Carl Gustav Carus Faculty of Medicine and University Hospital, Technische Universität Dresden, Dresden, Germany; 2 Department of Psychosomatic Medicine and Psychotherapy, Ulm University Medical Center, Ulm, Germany; 3 Department of Psychosomatics and Psychotherapy, University of Cologne, Medical Faculty and University Hospital, Cologne, Germany; 4 Department of Psychosomatic Medicine and Psychotherapy, University Clinic of Bonn, Bonn, Germany; 5 Department of Psychosomatic Medicine and Psychotherapy, University Hospital of Erlangen, Friedrich-Alexander University Erlangen-Nürnberg (FAU), Erlangen, Germany

**Keywords:** COVID-19 pandemic, fatherhood, fathers, healthcare workers, mental health, work–family conflict

## Abstract

**Background:**

The increased mental distress among healthcare workers (HCW) during the COVID-19 pandemic has been well documented. Frontline research has rarely focused on fathers among HCW. This web-based multi-center study aimed to evaluate work–family conflict (WFC) among HCW fathers during the COVID-19 pandemic.

**Methods:**

Work–family conflict was assessed in cross-sectional, partly overlapping subsamples of 2,844 fathers across four time points (T1: *n* = 1,155; T2: *n* = 930; T3: *n* = 343; T4: *n* = 416) from April 2020 to May 2022, and compared to 6,981 age-matched mothers and 1,194 male colleagues without children. A longitudinal subsample of *n* = 188 fathers who participated at two or more time points was analyzed separately. The impact of workload, exhaustion, fear, moral concerns, and institutional trust on HCW fathers’ WFC was analyzed using exploratory factor analysis, cross-sectional linear modeling, and longitudinal linear mixed-effects modeling.

**Results:**

Fathers reported higher WFC at T1–T3, with levels comparable to mothers, except at T2, when fathers exceeded them. WFC increased from T1 to T4. Risk factors included higher workload, exhaustion, moral concerns, and lower institutional trust, with exploratory longitudinal evidence pointing to a growing protective role of institutional trust against WFC over time. Younger age, cohabitation, children in the household, contact with COVID-19 and full-time employment were also linked to higher WFC. More fathers were working full-time than childless male colleagues.

**Conclusion:**

Our findings underscore that WFC among HCW fathers is a structural issue that requires targeted workplace adaptations during crises. Addressing fathers’ caregiving roles through flexible and culturally sensitive policies will be crucial to mitigate conflict and promote sustainable work–family integration. Future research should examine causal pathways to develop tailored work-life balance models for fathers.

## 1 Introduction

The COVID-19 pandemic caused sweeping disruptions to professional and private life worldwide, placing particular strain on families with children (1–3). In Germany, parents faced the simultaneous demands of remote work, childcare, and home-schooling while confined in close quarters (4). Two-thirds of parents reported difficulties balancing these demands, and reduced psychological well-being or increased negative affect was widely documented (5–8).

Work–family conflict (WFC) and Family–work conflict (FWC) arise when pressures from work and family are mutually incompatible, leading to stress, reduced commitment, diminished job-/family-, life satisfaction and well-being (9, 10). WFC has been shown to increase personal strain (10), contribute to mental and physical health problems (11), depression (12), emotional exhaustion (13), and burnout (14). Above, it is associated with increased sick leave and an economic and social burden (15).

The pandemic exacerbated the demands of fulfilling multiple roles. Lockdown measures intensified the collision between professional obligations and caregiving duties, particularly by increasing time- and strain-based conflicts (16, 17). Pre-pandemic studies found systematic differences in aspects of parental status and gender (18, 19), though results were mixed. For instance a large meta-analysis outlined more similarities than differences between men and women, with women experiencing slightly more conflict overall (17). Men and women seem to navigate the blurred boundaries between work and family differently There are hints of greater role segmentation and stronger boundary formation in men, but the results are also conflicting (17, 20). Women (rather than man) may experience internal conflict, guilt, and feelings of inadequacy when occupying dual roles, which increases stress and perceptions that roles are in conflict, particularly when work interferes with family (17). However, a meta-analytic review highlighted that gender plays a minor role and in particularly work factors relate more strongly to WFC (21). Structural inequalities, such as limited workplace flexibility and an unequal division of caregiving tasks, lead to greater difficulty maintaining work-life balance, in particular for employed mothers than for employed fathers (7, 22, 23). Recent literature emphasizes gender differences in work–family conflict in particular for HCW (24), an occupational group with well-known increased risk of developing mental health problems, both before and during the pandemic (25).

During the pandemic, HCW were often unable to work remotely. They had to cope with longer working hours and work-related trauma while facing dual stressors, comprising increased job demands and heightened caregiving responsibilities at home (26, 27). In particular HCW parents reported increased parental distress compared with other occupational groups (4, 16, 28). Above, HCW families’ mental health issues worsened (25). Besides the impact of the profession on WFC and parental distress, other factors that increase vulnerability for working parents include high education, low income, single status, female gender, and caring for children younger than 11 years old or children with chronic illnesses (4, 16, 29, 30).

While fathers have increasingly engaged in childrearing over the past decades (31–33), the pandemic accelerated this trend for some families, going along with shifts in paternal roles, leading to greater permeability between the family and work domain (30). One could hypothesize that working from home could promote involved fathering and stronger family relationships, potentially strengthening the family’s overall capacity to combat the stressors and challenges brought by the COVID-19 pandemic (29). With many men working from home or working part-time, studies noted increased paternal caregiving and participation in housework (34–37), though mothers continued to carry the larger share of domestic responsibilities (30, 38, 39). Although some couples reported more egalitarian divisions of labor (30), overall, gender asymmetries still persist, for instance in Germany (16).

Despite this evolution, fathers remain underrepresented in family research. Empirical studies often overlook male caregivers or associate parenting with mothering (32, 33). Calls have been made to embed fathers more systematically within family systems research to capture the full complexity of modern parenting – in particular in times of crises (40).

Existing research on WFC during COVID-19 remains fragmented, especially regarding gender differences, caregiving roles, and occupational stress, in particular in essential, frontline occupations such as health care work. Most studies were cross-sectional, limiting insights into how WFC evolves over time, sheeding light on influencing variables and causal mechanisms (29). Finally, there is meaningful variation in the magnitude of gender differences in WFC, but the key factors that determine this variation is currently not well understood, in particular with respect to work-related influences (17, 21). Furthermore, few studies have systematically analyzed the pandemic’s gendered consequences through the lens of blurred work-family boundaries (2), and those that do often neglect the unique burdens faced by HCW with caregiving responsibilities (41, 42). There remains limited research on the psychological burden of caregiving among mothers and fathers, particularly in essential sectors like healthcare (6, 43).

In occupational contexts, high workload, lack of flexibility, and insufficient support were established predictors of WFC (44–46). Social support at the workplace has been identified as a key buffer against WFC and decreased well-being (47), yet it was frequently lacking during the pandemic (48). Furthermore, perceived organizational support has been identified to be an important factor in managing WFC of nurses (49).

A growing consensus calls for longitudinal research to uncover cumulative and delayed effects and to clarify how occupational and psychosocial factors jointly shape WFC (29), and to deepen the knowledge about fathers’ engagement in care and part-time or full-time work (50). The present study addresses these gaps by investigating WFC among HCW, with a particular focus on HCW fathers. We aimed to examine WFC trajectories among HCW fathers compared to their childless male colleagues and HCW mothers during the COVID-19 pandemic and to explore the influence of occupational stressors, sociodemographic and psychosocial factors.

We hypothesize that perceived WFC increased during the COVID-19 pandemic, and that fathers experienced particularly pronounced burdens, compared to mothers and male colleagues without children. By illuminating gendered stress pathways in a high-risk occupational group, this study contributes to a more nuanced understanding of parenting in times of societal crisis.

## 2 Materials and methods

### 2.1 Participants and procedures

The present study is part of the prospective VOICE study, conducted as part of the egePan Unimed project ‘Development, testing, and implementation of regionally adaptive care structures and processes for evidence-based pandemic management’, coordinated by university medical institutions (51). The online survey examined stressors and resources among HCW throughout the COVID-19 pandemic. The total sample of the VOICE study (*N* = 23,256) included *n* = 8,067 HCW at the first time point (T1, 04.2020–07.2020), *n* = 7,190 HCW at T2 (11.2020–01.2021), *n* = 3,463 HCW at T3 (05.2021–07.2021) and *n* = 4,536 HCW at T4 (02.2022–05.2022). The sample included physicians, nurses, medical technical assistants, psychologists, and administrative staff. For this study, a total of 2.844 HCW fathers were assessed (T1, *n* = 1,155; T2, *n* = 930; T3, *n* = 343; T4, *n* = 416) and compared to 6,981 HCW mothers (T1, *n* = 2,979; T2, *n* = 1,988; T3, *n* = 884; T4, *n* = 1,130) and 1,194 male HCW without children (T1, *n* = 409; T2, *n* = 372; T3, *n* = 183; T4, *n* = 230). A subsample of *n* = 188 HCW fathers, who participated in at least two of the four time points, was analyzed longitudinally. Male HCW without children were included as a comparison group to isolate the contribution of parental status to WFC, recognizing that even in the absence of caregiving duties, HCW may experience inter-role conflict due to pandemic-related social isolation and workload (18, 52). To ensure demographic comparability across groups, mothers and childless male colleagues were matched to the father sample separately at each time point (T1–T4). Specifically, the age group distribution of fathers at each wave served as the reference structure. Comparison group samples were then drawn via proportional stratified random sampling, whereby the proportion of each age stratum in the father sample was used to determine the number of participants drawn from the corresponding age stratum in each comparison group. This procedure was applied independently at each time point, resulting in age-composition-matched subsamples of mothers and childless male colleagues for cross-group comparisons of work–family conflict and its predictors.

This study employed an open recruitment design, with participants recruited at each time point via institutional mailing lists and professional platforms. As participation was voluntary at each wave, the cross-sectional subsamples at T1–T4 are partly overlapping and partly independent. To avoid pseudoreplication in cross-sectional analyses, only the first participation of each participant was retained, ensuring that each participant contributed a single observation.

The survey was shared via mailing lists distributed to hospital staff, professional organizations, and through professional online platforms. The general inclusion criteria included a minimum age of 18 years, employment in the healthcare sector, residence or workplace in Germany, and sufficient German language skills. Data was collected via self-report online questionnaires and compared to comparison samples of HCW mothers and male HCW without children for each time point. The present study was registered in ClinicalTrials (DRKS-ID: DRKS00021268).

### 2.2 Measures

Self-reported *WFC* was measured as the primary outcome. Scales by Netemeyer et al. (53) originally incorporated five items for each of the two subscales: family-to-work conflict (FWC) and work-to-family conflict (WFC) of which the first two of each subscale were used here. However, a shortened version, based on the 4-item version of Breyer and Bluemke (54) with four items was used: “The demands of my work interfere with my home and family life,” “The amount of time my job takes up makes it difficult to fulfill family responsibilities”, “The demands of my family or spouse/partner interfere with work-related activities.”, “I have to put off doing things at work because of demands on my time at home.” The validated German-language instrument captures both directions of conflict: work interference with family (WFC; two items) and family interference with work (FWC; two items). In the present study, we combined the two directions (WFC and FWC) and adhered to the definition of overall WFC, which is understood as interrole conflict resulting from incompatible demands between work and family domains (55). This is consistent with Breyer and Bluemke’s scoring recommendation, which suggests calculating a total WFC score as the mean of all four items to reflect the overall degree of bi-directional role conflict (54). This aggregated scoring approach was chosen because the research question addresses general WFC burden among HCW fathers rather than bi-directional effects specifically, and because it reduces model complexity and increases acceptability in the context of a longitudinal pandemic survey with multiple assessment points and assessed variables. Internal consistency for the German sample was acceptable for the total scale (*α* = 0.72) as well as for the WFC (*α* = 0.73) and FWC subscales [*α* = 0.66; (54)]. In the present sample, internal consistency was good for the total scale (*α* = 0.87) as well as for the WFC subscale (*α* = 0.90) and acceptable for the FWC subscale (*α* = 0.70). The two subscales were substantially correlated (*r* = 0.72, *p* < 0.001), supporting the use of a total score reflecting overall bi-directional WFC, consistent with the scoring recommendation by Breyer and Bluemke (54). Each item was scored on a five-point Likert scale (0 = ‘do not agree at all’, 4 = ‘totally agree‘) and the scores of the four items were averaged (range: 0–4). Higher totals indicate greater WFC.

Work- and COVID-19-related variables were assessed with 19 self-developed items based on scales created during the 2009 H1N1 pandemic (56), and were also rated on a five-point Likert scale from 0 (‘strongly disagree’) to 4 (‘strongly agree’), referring to the past 2 weeks. Four work-related-, and twelve COVID-19-related items were considered. Examples are: “I work more than before the pandemic.,” “There is sufficient staff.” (reversed), “I feared COVID-19 infection.,” “I feared infecting relatives with COVID-19.”, “I can recover sufficiently during spare time.” (reversed), “I suffered from insomnia.,” “I feared having to decide who gets care and who does not.,” “I was burdened by the idea, that patients died without seeing their dependents again.,” “During difficult times at work I can rely on my colleagues.” or “I felt protected by local authorities.” To examine the dimensionality of the work- and COVID-19-related items, an exploratory factor analysis using maximum-likelihood extraction and varimax rotation was conducted with these items. For further details on the assessed variables and results (see Supplementary Table S1).

Generally, sociodemographic characteristics which have been suggested to be related to the conflicts between work and family, were age (57), working hours and family hours (58), total years of work experience (59), presence of children living at home and living with a partner (60), single parent status (61–63), working full or part-time (64, 65), COVID-19 infection among HCW (66), further caring for a sick or older adult relative (67). In the consequence of existing literature and according to general implications (68), we intended to include the following sociodemographic covariates into the investigation: age group, living alone, having children in the household, single-parent status, caregiving for relatives, employment status and contact with COVID-19.

### 2.3 Statistical analyses

All statistical analyses were conducted using SPSS Core 29 (92) and R Version 4.4.1 (69), using the R-packages lme4 (70), tidyr (71), haven (72), tidyverse (73), MuMIn (74), lmerTest (75), broom.mixed (76), ggplot2 (77), dplyr (78), r2glmm (79), and car (80). Descriptive statistics summarizing the study population included measures of central tendency, standard deviation, and frequency distributions. Differences between HCW fathers, mothers and childless males were assessed using ANOVA for continuous variables and *χ^2^*-tests for categorical variables. Games-Howell post-hoc tests were conducted for direct comparisons between groups and timepoints. To avoid re-answering bias, only the first participation of each participant was included in cross-sectional analyses. In order to address the substantial attrition and the risk of selective attrition to systematically bias the results, a dropout analysis comparing completers and non-completers for differences in sociodemographic or baseline psychosocial variables was conducted. Additionally, to examine potential compositional differences across the cross-sectional subsamples, sociodemographic and psychosocial characteristics of fathers were compared across time points. Structural demographic variables, including employment status, parental household composition, single-parent status, and caregiving for relatives, did not differ significantly across time points (all *p* > 0.05). Age group (*p* < 0.001) and living alone (*p* = 0.011) showed statistically significant but substantively small differences. Based on correlational analyses [Supplementary Table S2, all sociodemographic variables were included as covariates in the linear (mixed) models].

To account for multiple testing, we stringently applied Bonferroni-corrected *p*-values (*p*/number of tests), calculated separately for the *χ^2^-*tests for each sample investigated. As predictors were selected based on theoretical and empirical considerations rather than exploratory testing, no additional correction for multiple comparisons was applied to the regression coefficients (68). Statistical significance for all tests was set at *p* < 0.05.

Dropout analysis was conducted to examine systematic differences in sociodemographic factors and baseline variables between non-completers, participants who completed only T1 and (near-) completers, who also completed at least two other timepoints.

To examine the dimensionality of the work- and COVID-19-related items, we conducted an exploratory factor analysis using maximum-likelihood extraction and varimax rotation. Kaiser–Meyer–Olkin (KMO) values were calculated to assess sampling adequacy, and Bartlett’s test of sphericity was applied to evaluate factorability. The number of factors was determined using eigenvalues greater than 1. Items with factor loadings below 0.30 were excluded. According to Kaiser, a *KMO* > 0.90 is considered ‘marvelous’, values > 0.80 are ‘meritorious’, and values > 0.70 are ‘middling’. Additionally, correlations between the factors and WFC were calculated using Pearson correlations.

A linear model using maximum likelihood estimation was fitted to examine the effects of workload, exhaustion, fear, moral concerns, and institutional trust, together with relevant sociodemographic covariates (age-group, employment status, contact with COVID-19, single parent status, living alone, children inside/outside the household, caregiving for relatives) on WFC among fathers. Additionally, we conducted a linear mixed-effects model (LMM) to account for repeated measures and interindividual differences by including random intercepts for participants. Fixed effects comprised the five psychosocial predictors and their interactions with timepoint. Lagged effects were specified for the psychosocial predictors as well as for sociodemographic and COVID-19-related covariates (caregiving for relatives, living alone, children in the household, employment status, single-parent status, and contact with COVID-19). Based on the method proposed by Nakagawa and Schielzeth (81), the variance explained by the LMM was quantified using *R^2^ marginal* and *R^2^ conditional*. These metrics reflect the proportion of variance explained by fixed effects alone (*R^2^ marginal*) and by both fixed and random effects combined (*R^2^ conditional*). Following the recommendations by Rights and Sterba (82), *R^2^ marginal*-values in psychological research using multilevel models are typically small to moderate. Values > 0.30 indicate meaningful explanatory power of fixed effects. *R^2^ conditional* incorporates variance components that are not the primary focus of hypothesis testing and are sample-dependent. Therefore, the marginal *R^2^* was considered the primary indicator of model fit for evaluating the explanatory power. Semi-partial *R^2^* values for individual fixed effects within the LMM were calculated using the standardized generalized variance (83). As the present study constitutes a secondary analysis of the VOICE cohort, no a-priori sample size calculation was performed specifically for the father subgroup. To evaluate the statistical adequacy of the achieved sample sizes, sensitivity analyses were conducted (84). For the cross-sectional linear model (*n* = 2,150), the minimum detectable effect size with 80% power at *α* = 0.05 was *f^2^* = 0.004, indicating sufficient power to detect even very small effects. For the longitudinal LMM (*n* = 188), the minimum detectable effect size was *f^2^* = 0.050 (semi-partial *R^2^* = 0.047), corresponding to a small-to-moderate effect. This indicates adequate power for the key effects observed in this study, while acknowledging that smaller effects may have remained undetected. This indicates adequate power to detect the primary effects of interest, while smaller or subgroup-specific effects in the longitudinal analyses may have remained undetected.

## 3 Results

### 3.1 Description of the total study sample

Sociodemographic, work-, and COVID-19-related characteristics of the study sample are summarized in Table 1. Overall, fathers were more frequently working full-time than mothers and were less likely to be single parents. They were also less likely to live alone than male HCW without children and mothers. Fathers were less frequently responsible for caring for relatives outside the home compared to mothers, but more frequently caring for relatives than their male colleagues without children. All included variables significantly correlated with WFC at some point during the pandemic (*p* ≤ 0.05; see Supplementary Table S2).

**Table 1:** Characteristic of the study sample.

Characteristic		Fathers, *n* = 2.844	Mothers, *n* = 6.981	MWC, *n* = 1.194	*p* between		
					GEN	F/M	F/MWC
Age group, years, *n* (%)	18–30	64 (2.3)	150 (2.1)	48 (4.0)	**<0.001**	**<0.001**	**<0.001**
	31–40	594 (20.9)	1,546 (22.1)	455 (38.1)			
	41–50	779 (27.4)	2079 (29.8)	296 (24.8)			
	51–60	976 (34.3)	2,607 (37.3)	301 (25.2)			
	> 60	431 (15.2)	599 (8.6)	94 (7.9)			
Employment status, *n* (%)	Full-time	2.354 (84.6)	2,653 (38.6)	962 (80.6)	**<0.001**	**<0.001**	**0.003**
	Part-time	429 (15.4)	4,216 (61.4)	182 (15.2)			
	Missing	61 (2.1)	59 (1.5)	50 (4.2)			
Contact with COVID-19, *n* (%)	Yes	1.373 (48.3)	4,073 (58.3)	605 (50.7)	**<0.001**	**<0.001**	**<0.001**
	No	1.394 (49.0)	2,751 (39.4)	526 (44.1)			
	Missing	77 (2.7)	157 (2.2)	63 (5.3)			
Single parent, *n* (%)	Yes	80 (2.8)	1,142 (16.4)	–	-	**<0.001**	-
	No	2.764 (97.2)	5,838 (83.6)	–			
Living alone, *n* (%)	Yes	228 (8.0)	757 (10.8)	474 (39.7)	**<0.001**	**<0.001**	**<0.001**
	No	2.616 (92.0)	6,224 (89.2)	720 (60.3)			
Children, *n* (%)	Yes, own house	1.949 (68.5)	4,840 (69.3)	–	-	0.450	**-**
	Yes, different house	895 (31.5)	2,141 (30.7)	–			
	No	–	–	1,194 (100)			
Caregiving for relatives, *n* (%)	Yes, own house	119 (4.2)	299 (4.3)	32 (2.7)	**<0.001**	**<0.001**	**0.020**
	Yes, different house	358 (12.6)	1.179 (16.9)	126 (10.6)			
	No	2.367 (83.2)	5.503 (78.8)	1,036 (86.8)			

Significant *p*-values are bold; “–” = not applicable; MWC = male HCW without children, n = sample size; F/M = test between fathers and mothers; F/MWC = test between fathers and MWC; GEN = general. Subsamples at each time point are partly overlapping and partly independent due to the open recruitment design and attrition. Sociodemographic characteristics of fathers were statistically comparable across time points (all *p* > 0.05 for structural variables). Comparison groups (mothers, childless male colleagues) were age-matched to the father sample at each time point via proportional stratified sampling.

### 3.2 Dropout analysis

A dropout analysis was conducted to reveal potential systematic differences between participants who completed only T1 (*n* = 994) and those who also completed at least two other timepoints (*n* = 55). The analysis revealed no significant difference in any demographic or baseline psychosocial variables (see Supplementary Table S3). Demographic characteristics of the cross-sectional father subsamples were comparable across all time points.

### 3.3 Exploratory factor analysis

**Figure 1:**
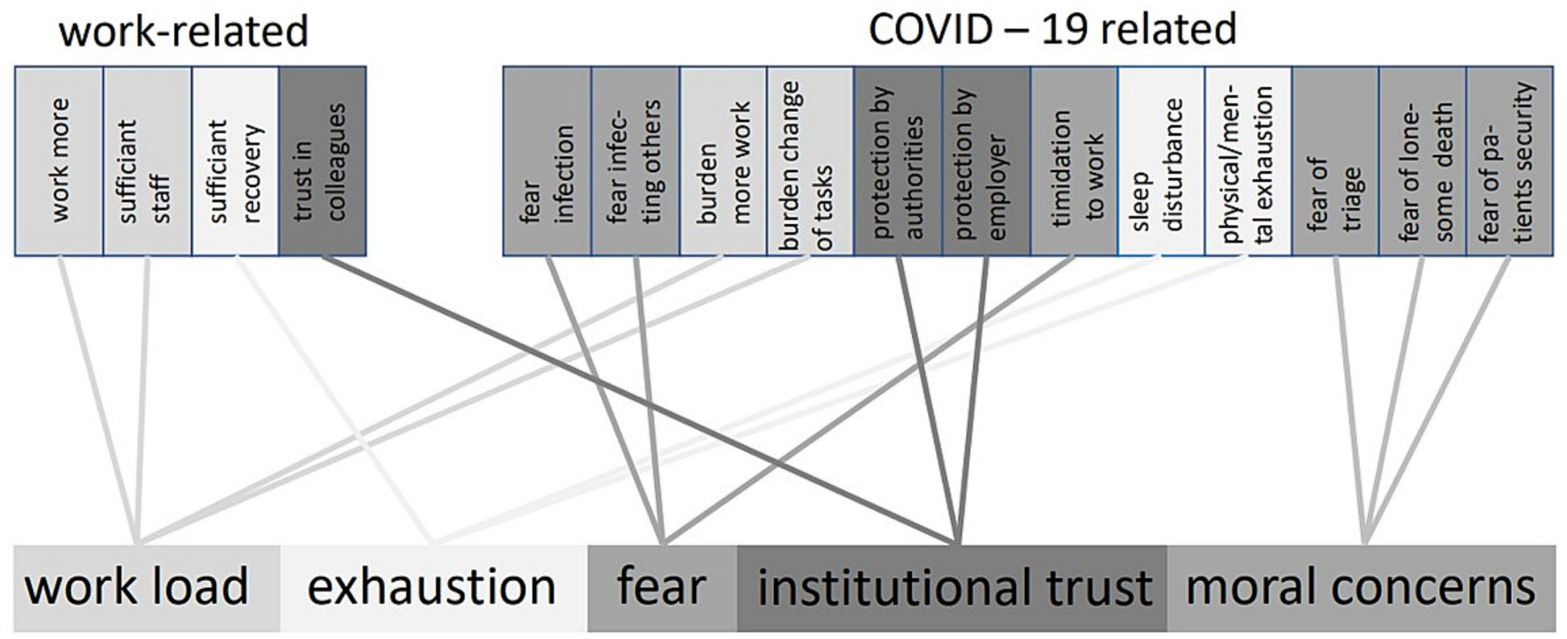
Visualization of the exploratory factor analysis, assigning 19 work- and COVID-19-related items to five overall influencing factors.

In the exploratory factor analysis, all individual KMO values were well above 0.70, with most exceeding 0.80, and the overall KMO was 0.82, confirming the adequacy of the data for factor analysis. Bartlett’s test of sphericity [*χ^2^* (*n* = 11.019) = 59331.427, *df* = 171, *p* < 0.001] indicated patterned relationships between the items. The final solution explained 41.92% of variance. Five factors were extracted: workload, exhaustion, fear, moral concerns and institutional trust (see Figure 1 and Supplementary Table S1). Pearson correlations between the factors and WFC are reported in Supplementary Table S4.

### 3.4 WFC of fathers compared to mothers and male HCW without children and its trajectory during the pandemic

**Figure 2:**
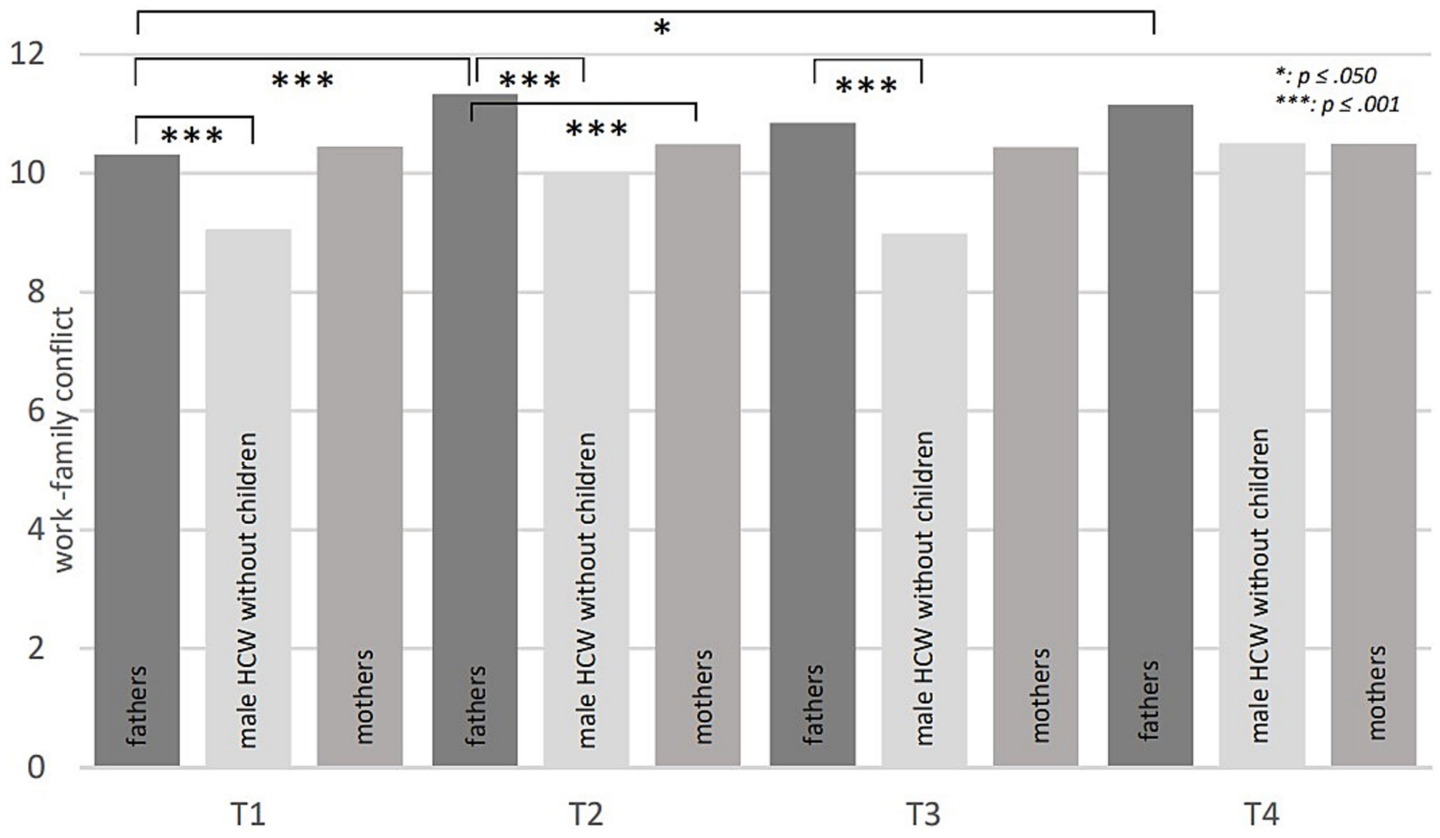
Work-family conflict of fathers compared with mothers and male HWC without children at T1, T2, T3, and T4.

The comparison in WFC between fathers, mothers, and male HCW without children throughout the COVID-19 pandemic is visualized in Figure 2. Full statistical results are provided in Supplementary Table S5. Fathers reported higher WFC than male HCW without children at T1 and T3, with levels comparable to mothers. At T2, fathers showed the highest WFC, exceeding both mothers and male HCW without children. By T4, no group differences were observed. Among fathers, WFC increased from T1 to T2 and from T1 to T4, but not from T1 to T3. A similar trajectory was observed for male HCW without children, whereas mothers showed no significant changes across all time points.

### 3.5 Workload, exhaustion, fear, moral concerns, and institutional trust of fathers compared to mothers and male HCW without children and their trajectory during the pandemic

The comparison and trajectory of workload, exhaustion, fear, moral concerns and institutional trust between fathers, mothers and male HCW without children throughout the COVID-19 pandemic is reported in Supplementary Table S5. At T1, fathers showed lower workload, exhaustion, fear, and moral concerns than mothers, and higher institutional trust than male HCW without children. At T2, fathers reported lower exhaustion and fear than mothers, while workload, moral concerns, and institutional trust were similar across groups. At T3, fathers displayed lower exhaustion than mothers, with fear, workload, moral concerns, and institutional trust showing similar levels across groups. At T4, fathers reported lower exhaustion and fear than both comparison groups. Workload was lower than in male HWC without children but similar to mothers. Moral concerns and institutional trust showed similar levels across groups.

**Figure 3:**
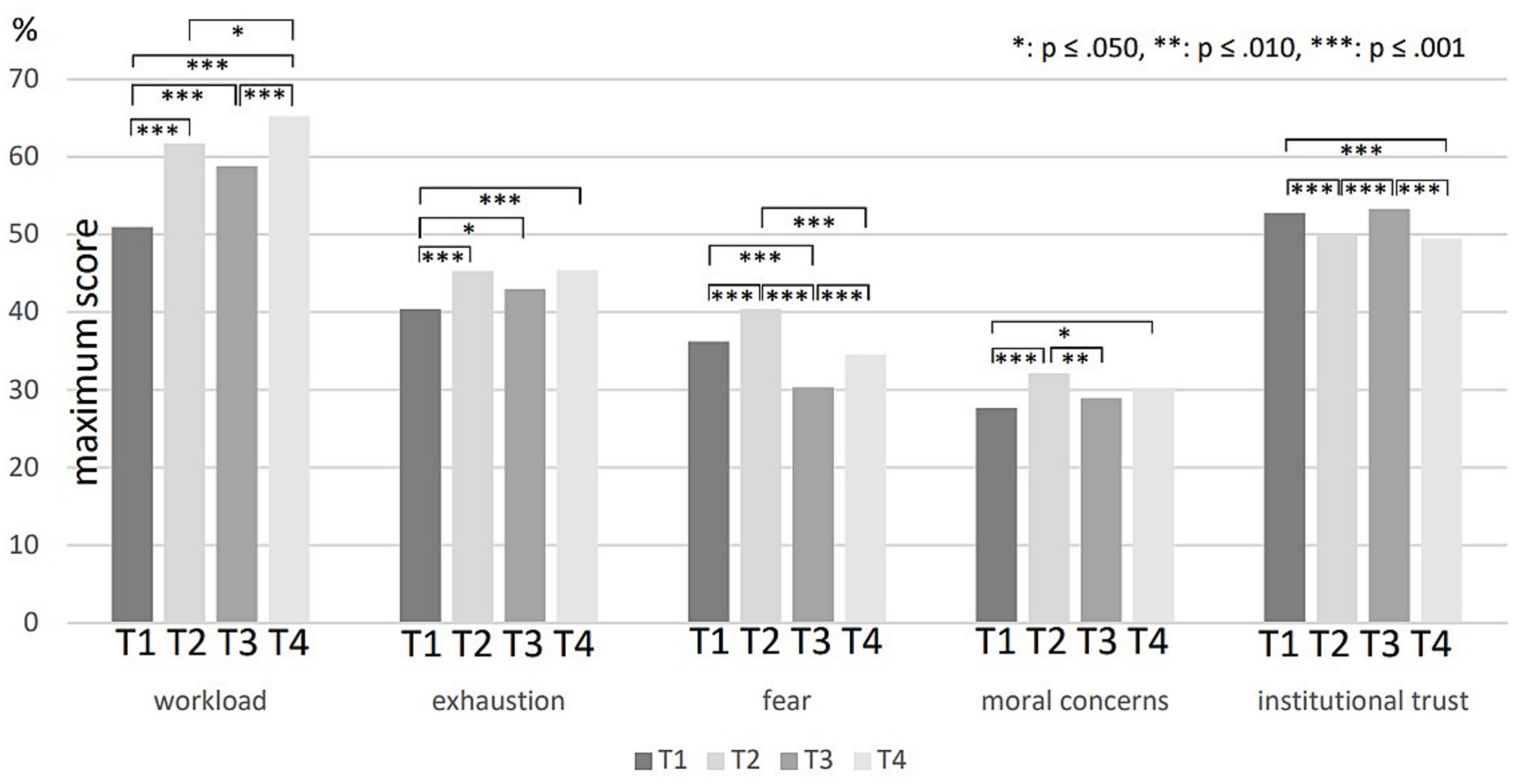
Workload, exhaustion, fear, moral concerns, and institutional trust of fathers at T1–T4.

The trajectory of workload, exhaustion, fear, moral concerns and institutional trust among fathers is visualized in Figure 3. All factors showed significant overall changes across time (all: *p* < 0.001). Workload and exhaustion rose from T1 to T2, dipped at T3, and increased again at T4. Fear increased from T1 to T2 but declined thereafter, with lower levels at T3 and T4. Moral concerns rose from T1 to T2, decreased at T3, and slightly increased again at T4. Institutional trust decreased from T1 to T2, peaked at T3, and declined again at T4. For full results (see Supplementary Table S5).

### 3.6 Linear model of WFC among HCW fathers: cross-sectional sample (*n* = 2,150)

A linear model of WFC, controlling for workload, exhaustion, fear, moral concerns, institutional trust and sociodemographic variables (age-group, living alone, employment status, caregiving for relatives, children inside/outside the household, single parent status, contact with COVID-19) confirmed multiple significant predictors for WFC (see Table 2). Higher levels of workload and exhaustion, and lower levels of institutional trust predicted higher WFC. Younger age, living with others, working full-time, having children in the household, and contact with COVID-19 were also associated with higher WFC. The adjusted *R^2^* of 0.435 indicated that the model explained approximately 43.5% of the variance in WFC. For full model results and diagnostics (see Supplementary Material S6).

**Table 2:** Fixed effects from the linear model of workload, exhaustion, fear, moral concerns and institutional trust on WFC of fathers: cross-sectional sample (*n* = 2,150).

Fixed effect	*b*	95%-CI	SE	*t*	*P*
Intercept	1.86	1.62, 2.10	0.13	14.91	<0.001***
Time point 2	−0.06	−0.14, 0.02	0.04	−1.39	0.164
Time point 3	−0.02	−0.12, 0.09	0.21	−0.29	0.774
Time point 4	−0.21	−0.32, −0.10	0.06	−3.77	<0.001***
Workload	0.07	0.05, 0.08	0.01	11.86	<0.001***
Exhaustion	0.11	0.01, 0.13	0.01	16.50	<0.001***
Fear	0.01	−0.00, 0.02	0.01	1.30	0.193
Moral concerns	0.01	−0.00, 0.03	0.01	1.75	0.081
Institutional trust	−0.02	−0.03, −0.00	0.01	−2.32	0.020*
Age group	−0.14	−0.17, −0.10	0.02	−6.78	<0.001***
Living alone	−0.16	−0.30, −0.02	0.07	−2.20	0.028*
Employment status	0.16	0.07, 0.26	0.05	3.42	<0.001***
Caregiving for relatives	0.06	−0.01, 0.13	0.04	1.71	0.088
Children in the own household	0.37	0.22, 0.40	0.05	6.56	<0.001***
Single parent	0.04	−0.17, 0.26	0.11	0.39	0.696
Contact with COVID-19	0.19	0.11, 0.26	0.04	5.06	<0.001***

95%-CI = confidence intervals (2.5, 97.5%); SE = standard error; b = unstandardized regression coefficient. Significant *p*-values are bold and marked: *p ≤ 0.050, ***p ≤ 0.001.

### 3.7 Linear mixed-effects model (LMM) of WFC among HCW fathers: longitudinal sample (*n* = 188)

An exploratory LMM of WFC was conducted (see Table 3), controlling for workload, exhaustion, fear, moral concerns, institutional trust and their interaction with timepoint and lagged values, as well as sociodemographic variables and their lagged values also confirmed multiple significant predictors. The main effects of workload, exhaustion, contact with COVID-19 and moral concerns were predictors for higher WFC. Lagged effects indicated that earlier contact with COVID-19 and full-time work predicted higher subsequent WFC. A significant negative interaction effect between timepoint and institutional trust suggests that institutional trust had an increasingly stronger negative impact on WFC as the pandemic progressed. The LMM explained 55.7% of the variance in WFC through fixed effects alone (*R^2^ marginal* = 0.56) and 92.0% when including random effects (*R^2^ conditional* = 0.92), indicating a strong model fit. Approximately 82.0% of the variance in WFC was attributable to between-participant differences in baseline WFC (*ICC* = 0.82). For full model results and diagnostics (see Supplementary Material S7).

**Table 3:** Fixed effects from the LMM of workload, exhaustion, fear, moral concerns, and institutional trust on WFC among fathers: longitudinal sample (*n* = 188).

Fixed effect	*b*	95%-CI	SE	df	*t*	*P*	Semi-partial *R*^2^
Intercept	1.45	0.62, 2.30	0.42	210.27	3.43	<0.001***	**–**
Time point	0.01	−0.09, 0.11	0.05	131.13	0.15	0.879	0.00
Workload	0.05	0.01, 0.10	0.02	147.71	2.36	0.020*	**0.02**
Exhaustion	0.06	0.00, 0.11	0.03	104.41	2.15	0.034*	**0.01**
Fear	0.05	−0.00, 0.10	0.03	159.81	1.89	0.061	0.01
Moral concerns	0.06	0.01, 0.10	0.02	147.73	2.56	0.012*	**0.02**
Institutional trust	0.02	−0.04, 0.08	0.03	150.21	0.73	0.467	0.00
Caregiving for relatives	0.03	−0.30, 0.35	0.66	222.62	0.16	0.870	0.00
Caregiving for relatives (lag)	0.04	−0.30, 0.38	0.70	220.95	0.22	0.828	0.00
Contact COVID-19	0.23	0.02, 0.44	0.43	182.66	2.15	0.033*	**0.02**
Contact COVID-19 (lag)	0.30	0.08, 0.51	0.44	175.19	2.72	0.007**	**0.03**
Age group	−0.12	−0.25, 0.01	0.26	209.11	−1.84	0.067	0.02
Workload (lag)	0.02	−0.02, 0.05	0.06	207.66	0.99	0.321	0.00
Exhaustion (lag)	0.04	−0.00, 0.08	0.08	180.35	1.83	0.069	0.01
Fear (lag)	0.02	−0.02, 0.05	0.08	192.13	0.75	0.456	0.00
Moral concerns (lag)	0.00	−0.04, 0.04	0.08	172.44	0.12	0.907	0.00
Institutional trust (lag)	0.04	−0.01, 0.09	0.10	191.79	1.72	0.088	0.01
Employment	−0.10	−0.44, 0.24	0.69	98.80	−0.60	0.553	0.00
Employment (lag)	0.37	−0.01, 0.73	0.72	99.24	2.03	0.045*	**0.01**
Children	0.22	−0.21, 0.65	0.87	113.04	0.99	0.322	0.00
Children (lag)	0.19	−0.22, 0.59	0.82	95.83	0.91	0.365	0.00
Living alone	0.31	−0.25, 0.86	1.13	222.38	1.08	0.282	0.01
Living alone (lag)	−0.28	−0.80, 0.25	1.06	146.99	−1.03	0.303	0.00
Single parent	−0.61	−1.22, 0.01	1.25	157.83	−1.94	0.054	0.01
Single parent (lag)	0.59	−0.03, 1.21	1.25	154.83	1.89	0.060	0.01
Time point × workload	0.01	−0.03, 0.04	0.06	162.60	0.34	0.732	0.00
Time point × exhaustion	0.02	−0.03, 0.06	0.09	139.38	0.76	0.447	0.00
Time point × fear	0.01	−0.03, 0.05	0.09	173.91	0.58	0.563	0.00
Time point × moral concerns	−0.04	−0.08, 0.00	0.09	190.62	−1.80	0.073	0.01
Time point × institutional trust	−0.07	−0.12, −0.02	0.10	149.22	−2.67	0.008**	**0.02**

95%-CI = confidence intervals (2.5, 97.5%); SE = standard error; b = unstandardized regression coefficient. Significant p-values are bold and marked: *p ≤ 0.050, **p ≤ 0.010, ***p ≤ 0.001.

## 4 Discussion

### 4.1 General discussion

Guided by prior research, we hypothesized that HCW fathers would differ in their WFC compared to mothers and male HCW without children, and that sociodemographic, COVID-19-related, and work-related factors would influence fathers’ WFC over time. Our results showed that WFC increased among fathers during the pandemic, with levels largely comparable to mothers but exceeding those of male HCW without children from T1 to T3 and peaking above mothers at T2. The suggested rise is in line with suggestions of a large systematic review, that, with the COVID-19 pandemic, some experiences and mental health issues of families were worsened (25). Longitudinally, younger age, full-time employment, children in the household, higher workload, exhaustion, and moral concerns were associated with higher levels of WFC. In addition, prior COVID-19 exposure and earlier full-time work status were related to greater WFC at subsequent time points. Institutional trust emerged as a growing protective factor as the pandemic progressed. Cross-sectionally, higher workload and exhaustion were confirmed as risk factors, and institutional trust as a protective factor. Additionally, fathers who were younger, living with others, had children in the household, were full-time employed, or had prior contact with COVID-19 were at increased risk of experiencing WFC.

Our findings, that fathers were significantly more likely to work full-time than mothers and slightly more likely to do so as childless men, reflect persistent workplace norms, where paternal caregiving remains underacknowledged (85). Despite formal access to flexible arrangements, fathers rarely adjust their work schedules for family needs (86, 87). The invisibility of fathers’ care roles, paired with social expectations to remain economically productive, may heighten internal pressure and thus contribute to WFC. Indeed, some fathers reported strong institutional trust early in the pandemic, potentially reinforcing their continued work engagement at the expense of family balance. Of note, perceived organizational support has been identified an important factor in managing WFC (49).

The sharp rise in WFC among fathers at T2 may reflect mounting childcare demands during school and daycare closures, combined with heightened expectations around paternal involvement. These shifts align with a broader ideological transition toward “new fatherhood” (85) and higher father engagement throughout the pandemic (34, 37), yet the mismatch between societal expectations and assumably limited perfectly fitting institutional support for fathers (e.g., from authorities and the employer) may be leaving fathers caught in inter-role conflict. Though, more studies are needed to deepen the knowledge about fathers’ engagement in care and part-time or full-time work (29, 50).

Notably, WFC among childless men rose during the pandemic, reaching parity with fathers by T4. This suggests that WFC was not exclusive to parents, but also affected HCW without caregiving duties, likely due to social isolation and reduced access to compensatory resources (52). Despite higher caregiving responsibilities, mothers’ WFC remained relatively stable. However, they reported greater exhaustion and fear, consistent with prior findings on gendered stress burdens (43, 88). These results support evidence that women had less opportunity to recover from work and were more likely to shoulder emotionally taxing tasks such as homeschooling and caregiving for relatives (26, 89). In contrast, fathers may have engaged more frequently in positive parenting experiences, potentially buffering perceived exhaustion while still increasing WFC. The fact that workload is a robust driver of work–family conflict (WFC), both cross-sectionally and longitudinally, confirms prior evidence linking long work hours and high job demands to inter-role conflict (44, 90, 91). COVID-19 exposure emerged as an additional stressor, underlining the psychological toll of infection risk and the fear of transmitting the virus to one’s family. Exhaustion was another consistent predictor for WFC. Fathers experienced comparable or greater WFC than mothers, indicating possible gendered coping differences or unacknowledged burden. Furthermore, moral concerns related to triage and patient safety contributed to WFC. Whether these reflect independent causal influences or a shared vulnerability profile remains an open question for future research.

### 4.2 Strengths and limitations

To our knowledge, this is one of the first surveys comparing the WFC in HCW fathers, mothers, and childless male colleagues, using both cross-sectional and longitudinal data. However, several limitations must be acknowledged. All data were self-reported, and although anonymity minimized social desirability bias, objective validation was not possible. The voluntary nature of participation may have introduced selection bias, potentially overrepresenting either highly burdened or highly resourceful individuals. Although the four-item Work–Family Conflict Scale provides a validated German-language measure, its psychometric properties have not been specifically established for HCW fathers or pandemic contexts. In the present sample, however, internal consistency exceeded the values reported for the German normative sample, suggesting adequate measurement quality in this specific population. Full measurement invariance across subgroups and time points could not be examined within the scope of the present study. The full 19-item version could not be administered due to feasibility constraints inherent to longitudinal online surveys during an active pandemic. Statistically significant differences in age group and living situation across time points indicate residual compositional heterogeneity in the cross-sectional subsamples, which may confound observed time trends despite statistical control. A key limitation concerns the substantial attrition across waves, resulting in a longitudinal subsample of only *n* = 188 fathers. Although the dropout analysis revealed no systematic differences between completers and non-completers with respect to sociodemographic or baseline psychosocial variables, selective attrition on unobserved variables cannot be ruled out. This degree of attrition fundamentally constrains the generalizability and reliability of longitudinal inferences. Time-trend interpretations, including the interaction between institutional trust and time, should therefore be regarded as preliminary and with caution. While the cross-sectional model afforded sufficient power to detect even very small effects (*f^2^* = 0.004), sensitivity analyses for the longitudinal LMM indicated a minimum detectable effect size of *f^2^* = 0.050, suggesting adequate power for the primary effects observed, while smaller or subgroup-specific effects may have remained undetected. Findings from the longitudinal analyses should therefore be regarded as exploratory and interpreted with caution. Short forms of validated scales were used to reduce burden, which may have affected construct validity.

Considering the modest number of observations compared to model complexity in the LMM, we applied parametric bootstrapping, which confirmed the stability of our estimates. Diagnostic checks supported model assumptions. Still, the model complexity risks overfitting and unstable estimates.

Another limitating point is, that the choice of time points was not theoretically derived but reflect the pragmatic structure of the VOICE study cohort, which constrains the identification of theoretically meaningful trajectories.

Finally, future research should aim for larger samples, higher measurement density, retention rates, and the inclusion of causal mediators to better understand the complex dynamics between occupational conditions, gender roles, and family demands during crises. Future studies should consider employing validated full-length instruments to strengthen measurement quality.

## 5 Conclusion

Our study demonstrates that HCW fathers experienced substantial WFC during the COVID-19 pandemic, exceeding that of childless men and, at times, even mothers. Our findings challenge assumptions of male psychological resilience in occupational stress research. Younger age, full-time employment, children in the household, high workload, exhaustion, and moral concerns contributed to WFC. Institutional trust emerged as an increasingly important protective factor over time, with direct implications for organizational interventions, especially regarding communication and supportive structures during crises. These findings underscore the need for structural and work schedule flexibility, culturally sensitive workplace policies, and targeted interventions to support evolving paternal roles and promote sustainable work-family integration for all caregivers.

## Data Availability

The raw data supporting the conclusions of this article will be made available by the authors, without undue reservation.
