# Supplementary material for: Fatherhood among healthcare workers: perceived work–family conflict and its influential factors during the COVID-19 pandemic: cross-sectional and exploratory longitudinal findings from the VOICE study
Source: Front Public Health. 2026 Jun 8;14:1782679. doi: 10.3389/fpubh.2026.1782679 (PMC13292114; doi:10.3389/fpubh.2026.1782679)
Supplement: Supplementary file 1 [file Supplementary_file_1.docx]

Supplementary material

**Table S1**. Description of work- and COVID-19-related variables and impact of these variables on the factor analysis

| variable abbreviation | Content | factor | loading |
| --- | --- | --- | --- |
| work. cond. 1 | There is sufficient protective gear. | ## |  |
| work. cond. 2 | I work more than before the pandemic. | 1 | .687 |
| work. cond. 3 (T1/T2) | T1: I work less than before the pandemic.  T2: I feel sufficiently informed about the pandemic. | ## |  |
| work. cond. 4 | There is sufficient staff. | (–) 1 ‘# | –.483 |
| work. cond. 5 | I can recover sufficiently during spare time. | (–) 3 # | –.504 |
| work. cond. 6 | During difficult times at work, I can rely on my colleagues. | 5 | .343 |
| work. cond. 7 (T2) ** | I feel better prepared than in spring. | ## |  |
| COVProb_1 | I feared COVID-19 infection. | 2 | .907 |
| COVProb_2 | I feared infecting relatives with COVID-19. | 2 | .731 |
| COVProb_3 | I felt burdened through increased workload. | 1 | .876 |
| COVProb_4 | I felt burdened through change of work tasks. | 1 | .418 |
| COVProb_5 | I felt protected through local authorities. | 5 | .512 |
| COVProb_6 | I felt protected through my employer. | 5 | .765 |
| COVProb_7 | I felt intimidated to work. | 2 | .449 |
| COVProb_8 | I suffered from insomnia. | 3 | .655 |
| COVProb_9 | I felt physically and mentally exhausted. | 3 | .763 |
| COVProb_10 | I feared having to decide who gets care and who doesn`t. | 4 | .403 |
| COVProb_11 | I was burdened by the idea, that patients died without seeing their dependents again. | 4 | .634 |
| COVProb_12 | I feared security of patients to be adversely affected. | 4 | .578 |
| COVProb_13 | I smoked more. | ### |  |
| COVProb_14 | I drank more alcohol. | ### |  |
| COVProb_15 | I took more antidepressants. | ### |  |
| COVProb_16 (T2) ** | I felt impaired due to omission of social contacts and spare time options. | ## |  |

*Note.* COVProb_1–16 = COVID-19-related risks and resources 1–16, work. cond. 1–7 = work-related risks and resources 1-7, T1 = time point 1, T2 = time point 2.

# Scale reversed.

##: excluded variable; not assessed at all time points.

###: excluded, by definition prior to analysis (coefficients < .3)

factor 1: “workload”, factor 2: “exhaustion”, factor 3: “fear”, factor 4: “moral concerns”, factor 5: “institutional trust”

**Table S2.** Correlation analyses of sociodemographic variables with WFC at T1, T2, T3, T4

| predictor | WFC | | | | | | |
| --- | --- | --- | --- | --- | --- | --- | --- |
|  | **T1** | **T2** | | **T3** | | **T4** | |
| age-group, *r* (*p*) [*n*] | **.281** (< .001) [1094] | | **.239** (< .001) [853] | | **–.374** (< .001) [343] | | **–.277** (< .001) [307] |
| living alone, *r* (*p*) [*n*] | .050 (.100) [1094] | | .044 (.196) [853] | | **.157** (.003) [343] | | .078 (.171) [307] |
| care for relatives, *r* (*p*) [*n*] | –.015 (.618) [1094] | | **–.069** (.043) [853] | | .042 (.436) [343] | | –.079 (.169) [307] |
| children, *r* (*p*) [*n*] | **–.244** (< .001) [1094] | | **–.187** (< .001) [853] | | **–.323** (< .001) [343] | | **–.284** (< .001) [307] |
| single parent, *r* (*p*) [*n*] | –.003 (.933) [1094] | | **–.071** (.038) [853] | | .001 (.986) [343] | | .074 (.194) [307] |
| employment, *r* (*p*) [*n*] | **–.063** (.038) [1094] | | **–.129** (< .001) [853] | | –.043 (.427) [343] | | **–.177** (.002) [307] |
| contact with COVID-19, *r* (*p*) [*n*] | **.086** (.004) [1093] | | **.233** (<.001) [853] | | **.182** (<.001) [343] | | **.252** (<.001) [307] |
| change of department, *r* (*p*) [*n*] | **–.084** (.006) [1094] | | **–.086** (.012) [853] | | –.075 (.163) [.343] | | **–.124** (.030) [307] |

*Note.* WFC = work–family conflict; *r* = Pearson correlation; *p* = *p*-value; *n* = sample size. Significant correlations are marked bold. Pearson-correlations.

**Table S3.** Dropout Analysis

| Characteristic |  | (Near-)Completers, *n* = 55^a^ | Dropout, *n* = 994^a^ | *p* |
| --- | --- | --- | --- | --- |
| age group | 18–30 | 1 (1.8%) | 22 (2.2%) | .439 |
|  | 31–40 | 7 (12.7%) | 191 (19.2%) |  |
|  | 41–50 | 18 (32.7%) | 257 (25.9%) |  |
|  | 51–60 | 23 (41.8%) | 357 (35.9%) |  |
|  | > 60 | 6 (10.9%) | 167 (16.8%) |  |
| employment | full-time | 47 (85.5%) | 829 (85.3%) | > .999 |
|  | part-time | 8 (14.5%) | 143 (14.7%) |  |
| single parent | Yes | 1 (1.8%) | 21 (2.1%) | > .999 |
|  | No | 54 (98.2%) | 973 (97.9%) |  |
| living alone | Yes | 7 (12.7%) | 60 (6.0%) | .079 |
|  | No | 48 (87.3%) | 934 (94.0%) |  |
| children | yes, own household | 35 (63.6%) | 677 (68.1%) | .553 |
|  | yes, other household | 20 (36.4%) | 317 (31.9%) |  |
|  | No | 0 (0.0%) | 0 (0.0%) |  |
| care for relatives | yes, own household | 0 (0.0%) | 36 (3.6%) | .419 |
|  | yes, other household | 6 (10.9%) | 125 (12.6%) |  |
|  | No | 49 (89.1%) | 833 (83.8%) |  |
| contact COVID-19 | yes | 31 (56.4%) | 465 (48.1%) | .268 |
|  | No | 24 (43.6%) | 502 (51.9%) |  |
| workload |  | 11.42 (3.98) | 10.06 (3.86) | .060 |
| exhaustion |  | 8.04 (2.76) | 8.03 (3.25) | .986 |
| fear |  | 6.55 (2.57) | 7.26 (3.07) | .108 |
| moral concerns |  | 5.24 (2.04) | 5.55 (2.45) | .600 |
| institutional trust |  | 11.04 (2.01) | 10.52 (2.45) | .286 |

*Note.* Significant *p*-values are bold.

^a^*n* (%); Mean (standard deviation)

**Table S4.** Correlation analyses between the factors of interest (influencing variables with work-family conflict/ WFC) at T1, T2, T3, T4

| Predictor | WFC | | | |
| --- | --- | --- | --- | --- |
|  | **T1** | **T2** | **T3** | **T4** |
| workload, *r* (*p*) [*n*] | **.476** (< .001) [894] | **.576** (< .001) [629] | **.438** (< .001) [343] | **.476** (< .001) [307] |
| exhaustion, *r* (*p*) [*n*] | **.538** (< .001) [894] | **.595** (< .001) [629] | **.483** (< .001) [343] | **.561** (< .001) [307] |
| fear, *r* (*p*) [*n*] | **.261** (< .001) [1094] | **.267** (< .001) [853] | **.187** (< .001) [342] | **.197** (< .001) [307] |
| moral concerns, *r* (*p*) [*n*] | .**262** (< .001) [1094] | **.330** (< .001) [853] | **.228** (< .001) [329] | **.282** (< .001) [307] |
| institutional trust, *r* (*p*) [*n*] | **–.240** (< .001) [886] | **–.329** (< .001) [629] | **–.137** (.011) [343] | **–.206** (< .001) [307] |

*Note.* WFC = work–family conflict; *r* = Pearson correlation; *p* = *p*-value; *n* = sample size. Significant correlations are marked bold. Pearson-correlations.

**Table S5.** WFC, workload, exhaustion, fear, moral concerns and institutional trust of fathers compared with mothers and male HCW without children at T1, T2, T3 and T4

|  |  |  | | | | | | | | | | | | ***p* within**^a,b^ | | | | | | | | | | | |  |
| --- | --- | --- | --- | --- | --- | --- | --- | --- | --- | --- | --- | --- | --- | --- | --- | --- | --- | --- | --- | --- | --- | --- | --- | --- | --- | --- |
|  | **group** | **T1** | | | **T2** | | | **T3** | | | **T4** | | | **AOV**^a^ | **T1-T2**^b^ | | **T1-T3**^b^ | | **T1-T4**^b^ | | **T2-T3**^b^ | | **T2-T4**^b^ | | **T3-T4**^b^ |  |
| **WFC,** *Mean (SD) [n]* | **F** | 10.31 (4.24) [1094] | | | 11.32 (4.34) [853] | | | 10.84 (3.96) [343] | | | 11.14 (4.17) [307] | | | **<.001** | **<.001** | | .143 | | **.012** | | .265 | | .919 | | .794 |  |
|  | **M** | 10.44 (4.49) [2800] | | | 10.48 (4.24) [1837] | | | 10.43 (4.02) [884] | | | 10.49 (4.11) [877] | | | .984 | .993 | | 1.000 | | .991 | | .993 | | 1.000 | | .990 |  |
|  | **MWC** | 9.05 (3.99) [359] | | | 9.98 (4.19) [324] | | | 8.98 (3.62) [183] | | | 10.50 (4.13) [179] | | | **<.001** | **.016** | | .997 | | **<.001** | | **.025** | | .533 | | **.001** |  |
|  |  | ***p* between**^a,b^ | | | | | | | | | | | |  |  | |  | |  | |  | |  | |  |  |
|  |  | **AOV^a^** | **F/M^b^** | **F/MWC^b^** | **AOV^a^** | **F/M^b^** | **F/MWC^b^** | **AOV^a^** | **F/M^b^** | **F/MWC^b^** | **AOV^a^** | **F/M^b^** | **F/MWC^b^** |  | |  | |  | |  | |  | |  | |  |
|  |  | **.001** | .664 | **.001** | **.001** | **.001** | **.001** | **.001** | .237 | **.001** | .056 | .051 | .236 |  | |  | |  | |  | |  | |  | |  |

*Note.* WFC = work-family conflict; *Mean* = arithmetic mean; *SD* = standard deviation; *n* = total sample; AOV = ANOVA; F = fathers; M = mothers; MWC = male healthcare workers without children.

Significance is marked bold. ^a^ANOVA. ^b^Games Howell Post hoc test.

|  |  |  | | | | | | | | | | | | ***p* within**^a,b^ | | | | | | | | | | |  |
| --- | --- | --- | --- | --- | --- | --- | --- | --- | --- | --- | --- | --- | --- | --- | --- | --- | --- | --- | --- | --- | --- | --- | --- | --- | --- |
|  | **group** | **T1** | | | **T2** | | | **T3** | | | **T4** | | | **AOV**^a^ | **T1-T2**^b^ | **T1-T3**^b^ | | **T1-T4**^b^ | | **T2-T3**^b^ | | **T2-T4**^b^ | | **T3-T4**^b^ |  |
| **wl,** *Mean (SD) [n]* | **F** | 10.18 (3.90) [903] | | | 12.35 (4.13) [638] | | | 11.75 (4.00) [343] | | | 13.04 (3.79) [313] | | | <.001 | **<.001** | **<.001** | | **<.001** | | .126 | | **.048** | | **<.001** |  |
|  | **M** | 10.58 (3.81) [2453] | | | 12.35 (4.13) [1482] | | | 11.78 (4.11) [881] | | | 13.17 (3.82) [894] | | | **<.001** | **<.001** | **<.001** | | **<.001** | | **.005** | | **<.001** | | **<.001** |  |
|  | **MWC** | 10.56 (3.95) [293] | | | 12.49 (4.08) [235] | | | 11.53 (3.62) [183] | | | 14.03 (4.01) [186] | | | **<.001** | **<.001** | **.031** | | **<.001** | | .053 | | **<.001** | | **<.001** |  |
|  |  | ***p* between**^a,b^ | | | | | | | | | | | |  |  |  | |  | |  | |  | |  |  |
|  |  | **AOV^a^** | **F/M^b^** | **F/MWC^b^** | **AOV^a^** | **F/M^b^** | **F/MWC^b^** | **AOV^a^** | **F/M^b^** | **F/MWC^b^** | **AOV^a^** | **F/M^b^** | **F/MWC^b^** |  |  | |  | |  | |  | |  | |  |
|  |  | **.024** | **.020** | .326 | .871 | 1.000 | .885 | .746 | .994 | .795 | **.012** | .862 | **.020** |  |  | |  | |  | |  | |  | |  |

*Note.* WL = workload; *Mean* = arithmetic mean; *SD* = standard deviation; *n* = total sample; AOV = ANOVA; F = fathers; M = mothers; MWC = male healthcare workers without children.

Significance is marked bold. ^a^ANOVA. ^b^Games Howell Post hoc test.

|  |  |  | | | | | | | | | | | | ***p* within**^a,b^ | | | | | | | | | | |  |
| --- | --- | --- | --- | --- | --- | --- | --- | --- | --- | --- | --- | --- | --- | --- | --- | --- | --- | --- | --- | --- | --- | --- | --- | --- | --- |
|  | **group** | **T1** | | | **T2** | | | **T3** | | | **T4** | | | **AOV**^a^ | **T1-T2**^b^ | **T1-T3**^b^ | | **T1-T4**^b^ | | **T2-T3**^b^ | | **T2-T4**^b^ | | **T3-T4**^b^ |  |
| **exh,** *Mean (SD) [n]* | **F** | 8.07 (3.27) [903] | | | 9.05 (3.32) [638] | | | 8.59 (2.94) [343] | | | 9.07 (3.17) [313] | | | **<.001** | **<.001** | **.035** | | **<.001** | | .117 | | .999 | | .176 |  |
|  | **Mean** | 8.86 (3.16) [2451] | | | 9.58 (3.08) [1482] | | | 9.37 (3.11) [884] | | | 9.75 (3.01) [894] | | | **<.001** | **<.001** | **<.001** | | **<.001** | | .353 | | .558 | | **.040** |  |
|  | **MWC** | 8.52 (3.46) [293] | | | 9.41 (3.19) [235] | | | 8.66 (3.19) [183] | | | 9.92 (3.05) [186] | | | **<.001** | **.012** | **.969** | | **<.001** | | .080 | | .330 | | **<.001** |  |
|  |  | ***p* between**^a,b^ | | | | | | | | | | | |  |  |  | |  | |  | |  | |  |  |
|  |  | **AOV^a^** | **F/M^b^** | **F/MWC^b^** | **AOV^a^** | **F/M^b^** | **F/MWC^b^** | **AOV^a^** | **F/M^b^** | **F/MWC^b^** | **AOV^a^** | **F/M^b^** | **F/MWC^b^** |  |  | |  | |  | |  | |  | |  |
|  |  | **.001** | **.001** | .123 | **.002** | **.001** | .305 | **.001** | **.001** | .967 | **.001** | **.003** | **.009** |  |  | |  | |  | |  | |  | |  |

*Note.* Exh = exhaustion; *Mean* = arithmetic mean; *SD* = standard deviation; *n* = total sample; AOV = ANOVA; F = fathers; M = mothers; MWC = male healthcare workers without children.

Significance is marked bold. ^a^ANOVA. ^b^Games Howell Post hoc test.

|  |  |  | | | | | | | | | | | | ***p* within**^a,b^ | | | | | | | | | | |  |
| --- | --- | --- | --- | --- | --- | --- | --- | --- | --- | --- | --- | --- | --- | --- | --- | --- | --- | --- | --- | --- | --- | --- | --- | --- | --- |
|  | **group** | **T1** | | | **T2** | | | **T3** | | | **T4** | | | **AOV**^a^ | **T1-T2**^b^ | **T1-T3**^b^ | | **T1-T4**^b^ | | **T2-T3**^b^ | | **T2-T4**^b^ | | **T3-T4**^b^ |  |
| **fear,** *Mean (SD) [n]* | **F** | 7.23 (3.04) [1105] | | | 8.08 (3.04) [865] | | | 6.06 (2.66) [342] | | | 6.89 (2.83) [313] | | | **<.001** | **<.001** | **<.001** | | .265 | | **<.001** | | **<.001** | | **<.001** |  |
|  | **M** | 7.89 (3.07) [2840] | | | 8.59 (3.01) [1859] | | | 6.38 (2.77) [880] | | | 7.70 (3.07) [894] | | | **<.001** | **<.001** | **<.001** | | .357 | | **<.001** | | **<.001** | | **<.001** |  |
|  | **MWC** | 7.52 (2.88) [367] | | | 8.29 (2.93) [329] | | | 5.93 (2.71) [183] | | | 7.95 (2.94) [186] | | | **<.001** | **.003** | **<.001** | | .369 | | **<.001** | | .582 | | **<.001** |  |
|  |  | ***p* between**^a,b^ | | | | | | | | | | | |  |  |  | |  | |  | |  | |  |  |
|  |  | **AOV^a^** | **F/M^b^** | **F/MWC^b^** | **AOV^a^** | **F/M^b^** | **F/MWC^b^** | **AOV^a^** | **F/M^b^** | **F/MWC^b^** | **AOV^a^** | **F/M^b^** | **F/MWC^b^** |  |  | |  | |  | |  | |  | |  |
|  |  | **.001** | **.001** | .224 | **.001** | **.001** | .536 | **.046** | .139 | .876 | **.001** | **.001** | **.001** |  |  | |  | |  | |  | |  | |  |

*Note.* *Mean* = arithmetic mean; *SD* = standard deviation; *n* = total sample; AOV = ANOVA; F = fathers; M = mothers; MWC = male healthcare workers without children.

Significance is marked bold. ^a^ANOVA. ^b^Games Howell Post hoc test.

|  |  |  | | | | | | | | | | | | ***p* within**^a,b^ | | | | | | | | | | |  |
| --- | --- | --- | --- | --- | --- | --- | --- | --- | --- | --- | --- | --- | --- | --- | --- | --- | --- | --- | --- | --- | --- | --- | --- | --- | --- |
|  | **group** | **T1** | | | **T2** | | | **T3** | | | **T4** | | | **AOV**^a^ | **T1-T2**^b^ | **T1-T3**^b^ | | **T1-T4**^b^ | | **T2-T3**^b^ | | **T2-T4**^b^ | | **T3-T4**^b^ |  |
| **MC,** *Mean (SD) [n]* | **F** | 5.52 (2.43) [1103] | | | 6.42 (2.86) [864] | | | 5.78 (2.70) [329] | | | 6.03 (2.66) [312] | | | **<.001** | **<.001** | .370 | | **.013** | | **.002** | | .121 | | .665 |  |
|  | **M** | 5.82 (2.65) [2833] | | | 6.36 (2.91) [1855] | | | 5.82 (2.80) [855] | | | 6.39 (2.93) [891] | | | **<.001** | **<.001** | 1.000 | | **<.001** | | **<.001** | | .997 | | **<.001** |  |
|  | **MWC** | 5.74 (2.73) [364] | | | 6.57 (3.09) [328] | | | 5.70 (2.55) [176] | | | 6.37 (2.84) [185] | | | **<.001** | **<.001** | .998 | | .064 | | **.004** | | .880 | | .088 |  |
|  |  | ***p* between**^a,b^ | | | | | | | | | | | |  |  |  | |  | |  | |  | |  |  |
|  |  | **AOV^a^** | **F/M^b^** | **F/MWC^b^** | **AOV^a^** | **F/M^b^** | **F/MWC^b^** | **AOV^a^** | **F/M^b^** | **F/MWC^b^** | **AOV^a^** | **F/M^b^** | **F/MWC^b^** |  |  | |  | |  | |  | |  | |  |
|  |  | **.005** | **.002** | .331 | .472 | .871 | .724 | .886 | .983 | .943 | .118 | .111 | .369 |  |  | |  | |  | |  | |  | |  |

*Note.* MC = moral concerns; *Mean* = arithmetic mean; *SD* = standard deviation; *n* = total sample; AOV = ANOVA; F = fathers; M = mothers; MCW = male healthcare workers without children.

Significance is marked bold. ^a^ANOVA. ^b^Games Howell Post hoc test.

|  |  |  | | | | | | | | | | | | ***p* within**^a,b^ | | | | | | | | | | |  |
| --- | --- | --- | --- | --- | --- | --- | --- | --- | --- | --- | --- | --- | --- | --- | --- | --- | --- | --- | --- | --- | --- | --- | --- | --- | --- |
|  | **Group** | **T1** | | | **T2** | | | **T3** | | | **T4** | | | **AOV**^a^ | **T1-T2**^b^ | **T1-T3**^b^ | | **T1-T4**^b^ | | **T2-T3**^b^ | | **T2-T4**^b^ | | **T3-T4**^b^ |  |
| **IT,** *Mean (SD) [n]* | **F** | 10.55 (2.43) [1103] | | | 9.97 (2.46) [637] | | | 10.65 (2.37) [343] | | | 9.89 (2.38) [313] | | | **<.001** | **<.001** | .904 | | **<.001** | | **<.001** | | .963 | | **<.001** |  |
|  | **M** | 10.41 (2.41) [2433] | | | 9.85 (2.39) [1482] | | | 10.44 (2.32) [884] | | | 9.95 (2.47) [894] | | | **<.001** | **<.001** | **<.001** | | **<.001** | | **<.001** | | .766 | | **<.001** |  |
|  | **MWC** | 9.92 (2.52) [289] | | | 9.98 (2.65) [235] | | | 10.43 (2.31) [183] | | | 9.56 (2.47) [185] | | | **.011** | .992 | .112 | | .418 | | .263 | | .325 | | **.003** |  |
|  |  | ***p* between**^a,b^ | | | | | | | | | | | |  |  |  | |  | |  | |  | |  |  |
|  |  | **AOV^a^** | **F/M^b^** | **F/MWC^b^** | **AOV^a^** | **F/M^b^** | **F/MWC^b^** | **AOV^a^** | **F/M^b^** | **F/MWC^b^** | **AOV^a^** | **F/M^b^** | **F/MWC^b^** |  |  | |  | |  | |  | |  | |  |
|  |  | **.001** | .329 | **.001** | .509 | .566 | .998 | .336 | .983 | .943 | .118 | .923 | .302 |  |  | |  | |  | |  | |  | |  |

*Note.* IT = institutional trust; *M* = arithmetic mean; *SD* = standard deviation; *n* = total sample; AOV = ANOVA; F = fathers; M = mothers; MWC = male healthcare workers without children.

Significance is marked bold. ^a^ANOVA. ^b^Games Howell Post hoc test

Supplement S6. Model selection process of the LM: cross-sectional analysis

The model selection process for the cross-sectional analysis was designed to examine the relationship between WFC and its potential predictors across all time points. This approach ensured transparency and methodological rigor, balancing theoretical considerations with statistical diagnostics.

**model specification.** The model included effects to capture the relationships between time, sociodemographic, psychological and work-related predictors on WFC. Predictors were selected based on theoretical frameworks and prior empirical findings that emphasized their relevance to WFC. The model included workload, exhaustion, fear, moral concerns, institutional trust, and demographic or situational variables such as age group, contact with COVID-19, living alone, caregiving responsibilities for other relatives, employment, children in the own household, and single parenthood.

To improve the interpretability of the regression coefficients, predictors such as workload, exhaustion, fear, moral concerns, institutional trust and age-group were mean-centered. This standardization ensured that regression coefficients reflected the relationship between each predictor and WFC at the mean value of the other predictors. Centering also minimized potential multicollinearity between interaction terms and their constituent variables, providing more stable and interpretable parameter estimates.

| **residual standard error** | .80 on 2134 degrees of freedom (694 observations deleted due to missingness) |
| --- | --- |
| **adj. *R²*** | .435 |
| ***F*-statistic** | 111.4 on 15 and 2134 degrees of freedom |
| ***P*** | **< .001***** |

| **residuals** | | | | |
| --- | --- | --- | --- | --- |
| **minimum** | **1^st^ quartile (25%)** | **Median** | **3^rd^ quartile (75%)** | **maximum** |
| –2.73 | –.55 | –.03 | .50 | 3.02 |

| fixed effect | *B* | *95%-CI* | *SE* | *t* | *p* |
| --- | --- | --- | --- | --- | --- |
| Intercept | 1.86 | 1.62, 2.10 | .13 | 14.91 | **< .001***** |
| time point 2 | –0.06 | –.14, .02 | .04 | –1.39 | .164 |
| time point 3 | –0.02 | –.12, .09 | .21 | –0.29 | .774 |
| time point 4 | –0.21 | –.32, –.10 | .06 | –3.77 | **< .001***** |
| Workload | 0.07 | .05, .08 | .01 | 11.86 | **< .001***** |
| Exhaustion | .11 | .01, .13 | .01 | 16.50 | **< .001***** |
| Fear | .01 | –.00, .02 | .01 | 1.30 | .193 |
| moral concerns | .01 | –.00, .03 | .01 | 1.75 | .081 |
| institutional trust | –.02 | –.03, –.00 | .01 | –2.32 | .**020*** |
| age group | –.14 | –.17, –.10 | .02 | –6.78 | **< .001***** |
| living alone | –.16 | –.30, –.02 | .07 | –2.20 | **.028*** |
| employment status | .16 | .07, .26 | .05 | 3.42 | **< .001***** |
| caregiving for relatives | .06 | –.01, .13 | .04 | 1.71 | .088 |
| children in the own household | .37 | .22, .40 | .05 | 6.56 | **< .001***** |
| single parent | .04 | –.17, .26 | .11 | .39 | .696 |
| contact with COVID-19 | .19 | .11, .26 | .04 | 5.06 | **< .001***** |

*Note.* 95%-*CI* = confidence intervals (2.5%, 97.5%); *SE* = standard error; *b* = unstandardized regression coefficient; adj*. R²* = adjusted coefficient of determination. Significant *p*-values are bold and marked: *: *p* ≤ .050, **: *p* ≤ .010, ***: *p* ≤ .001.

All categorical predictors were effect-coded as follows to aid interpretation of coefficient signs: Age group: reference = 18–30 (1), levels increasing up to >60 (5); Employment status: 0 = part-time, 1 = full-time; Contact with COVID-19: 0 = no, 1 = yes; Single parent: 0 = no, 1 = yes; Living alone: 0 = no, 1 = yes; Children: 0 = no, 1 = yes, but not in same household, 2 = yes, and in the same household; Caregiving for relatives: 0 = no, 1 = yes, but not in same household, 2 = yes, and in the same household. The first category of each categorial factor served as the reference group and is reflected in the intercept.

**model diagnostics.** Multicollinearity among predictors was evaluated using VIF-values. All predictors had values below 1.75, confirming tolerable levels of multicollinearity.

Residual diagnostics confirmed the suitability of the model. Normality of residuals was supported by a Kolmogorov-Smirnov test (*D* = .03, *p* = .109), which supported the null hypothesis of normality. Visual inspection of residuals revealed no substantial deviations from normality (see Figure A). Homoscedasticity was also validated through this plot, indicating consistent variance across the range of fitted values. The final model achieved a multiple *R²* of .439, indicating that approximately 43.9% of the variance in WFC was explained by the fixed effects. The adjusted *R²* of .435 confirmed the robustness of the model after accounting for the number of predictors. An F-statistic of 111.4 (*p* < .001) highlighted the overall significance of the model in explaining WFC variability.

**Figure A.** Q-Q Plot of the Residuals from the Linear Model
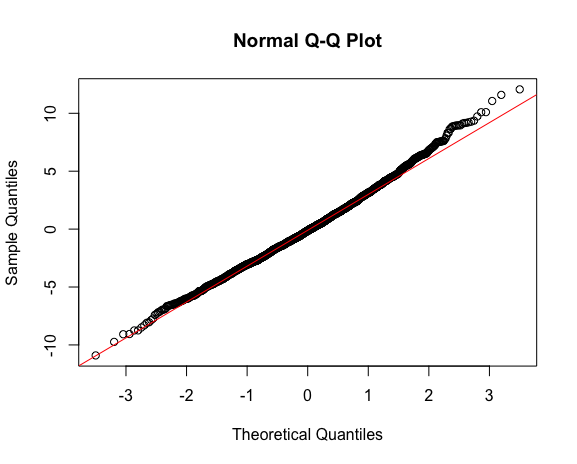


**Conclusion.** This cross-sectional model selection process empathized theoretical justification and empirical validation, resulting in a robust model that met all assumptions for linear regression. The strong model fit confirmed the relevance of the predictors in explaining WFC. The decision to center variables ensured interpretable coefficients and allowed for a clearer understanding of the relationships between the predictors and WFC.

**Supplement S7. Model selection process of the LMM: longitudinal analysis**

To ensure a transparent and replicable analysis, we carefully documented each step of our model selection process for the LMM. This process reflects both theoretical considerations and empirical diagnostics, aimed at capturing the temporal dynamics and variability in our data.

**model specification.** The model is a random intercept model, designed to account for inter-individual variability. ‘Participant Code’ was included as a random effect to control for participant-specific differences in baseline WFC. The fixed effects included a range of predictors hypothesized to influence WFC, such as workload, exhaustion, fear, moral concerns, and institutional trust. Timepoint was included as a fixed effect to model changes over the measurement points. Predictors were selected based on theoretical frameworks and prior empirical evidence suggesting their relevance in explaining variability in WFC.

The ICC was .82, indicating substantial between-participant clustering. Accordingly, the LMM included a random intercept for participants. Time (T1–T4) was modeled as a fixed effect. Allowing random slopes of time by participant did not improve fit (variance ≈ 0 / non-significant LRT), so the final specification was random-intercept–only. All predictors were modeled as fixed effects. This decision reflects the balance between empirical evidence and the need for a parsimonious model structure.

Predictors were centered where applicable to facilitate interpretation and reduce potential multicollinearity–particularly important considering the interaction effects involving continuous variables. Centering ensures that the intercept represents the predicted value of WFC when all predictors are at their average value, making the results more interpretable.

Interactions between timepoints and predictors were tested to investigate whether the effect of predictors varied across measurement points. The inclusion of these interaction terms allowed the model to account for time-dependent dynamics and provided insights into how the impact of predictors varied in influencing WFC.

To capture delayed effects of predictors on WFC, lagged versions of key variables were incorporated into the model. Including lagged predictors allowed us to explore how past states of predictors influenced WFC at later time points.

| ***AIC*** | ***BIC*** | **logLik** | **deviance** | ***df* residuals** |
| --- | --- | --- | --- | --- |
| 514.6 | 623.6 | -225.3 | 450.6 | 191 |

| **scaled residuals** | | | | |
| --- | --- | --- | --- | --- |
| **minimum** | **1st quartile (25%)** | **Median** | **3rd quartile (75%)** | **maximum** |
| –1.60 | –.29 | < .01 | .32 | 1.65 |

| **random effects** | | | |
| --- | --- | --- | --- |
| **groups** | **Variance** | ***SD*** | ***CI* 2.5%, 97.5%** |
| partipicants_ID (Intercept) | .43 | .66 | .56, .76 |
| residual | .10 | .31 | .24, .41 |

| fixed effect | *B* | 95%*-CI* | *SE* | *df* | *t* | *p* | *semi-partial R2* |
| --- | --- | --- | --- | --- | --- | --- | --- |
| intercept | 1.45 | .62, 2.30 | .42 | 210.27 | 3.43 | **< .001***** | **–** |
| time point | .01 | –.09, .11 | .05 | 131.13 | .15 | .879 | .00 |
| workload | .05 | .01, .10 | .02 | 147.71 | 2.36 | **.020*** | **.02** |
| exhaustion | .06 | .00, .11 | .03 | 104.41 | 2.15 | **.034*** | **.01** |
| fear | .05 | –.00, .10 | .03 | 159.81 | 1.89 | .061 | .01 |
| moral concerns | .06 | .01, .10 | .02 | 147.73 | 2.56 | **.012*** | **.02** |
| institutional trust | .02 | –.04, .08 | .03 | 150.21 | .73 | .467 | .00 |
| caregiving for relatives | .03 | –.30, .35 | .66 | 222.62 | .16 | .870 | .00 |
| caregiving for relatives_lag | .04 | –.30, .38 | .70 | 220.95 | .22 | .828 | .00 |
| contact COVID-19 | .23 | .02, .44 | .43 | 182.66 | 2.15 | **.033*** | **.02** |
| contact COVID-19_lag | .30 | .08, .51 | .44 | 175.19 | 2.72 | **.007**** | **.03** |
| age group | –.12 | –.25, .01 | .26 | 209.11 | –1.84 | .067 | .02 |
| workload_lag | .02 | –.02, .05 | .06 | 207.66 | .99 | .321 | .00 |
| exhaustion_lag | .04 | –.00, .08 | .08 | 180.35 | 1.83 | .069 | .01 |
| fear_lag | .02 | –.02, .05 | .08 | 192.13 | .75 | .456 | .00 |
| moral concerns_lag | .00 | –.04, .04 | .08 | 172.44 | .12 | .907 | .00 |
| institutional trust_lag | .04 | –.01, .09 | .10 | 191.79 | 1.72 | .088 | .01 |
| employment | –.10 | –.44, .24 | .69 | 98.80 | –.60 | .553 | .00 |
| employment_lag | .37 | –.01, .73 | .72 | 99.24 | 2.03 | **.045*** | **.01** |
| children | .22 | –.21, .65 | .87 | 113.04 | .99 | .322 | .00 |
| children_lag | .19 | –.22, .59 | .82 | 95.83 | .91 | .365 | .00 |
| living alone | .31 | –.25, .86 | 1.13 | 222.38 | 1.08 | .282 | .01 |
| living alone_lag | -.28 | –.80, .25 | 1.06 | 146.99 | -1.03 | .303 | .00 |
| single parent | -.61 | –1.22, .01 | 1.25 | 157.83 | -1.94 | .054 | .01 |
| single parent_lag | .59 | –.03, 1.21 | 1.25 | 154.83 | 1.89 | .060 | .01 |
| time point × workload | .01 | –.03, .04 | .06 | 162.60 | .34 | .732 | .00 |
| time point × exhaustion | .02 | –.03, .06 | .09 | 139.38 | .76 | .447 | .00 |
| time point × fear | .01 | –.03, .05 | .09 | 173.91 | .58 | .563 | .00 |
| time point × moral concerns | –.04 | -.08, .00 | .09 | 190.62 | –1.80 | .073 | .01 |
| time point × institutional trust | –.07 | –.12, –.02 | .10 | 149.22 | –2.67 | **.008**** | **.02** |

*Note.* 95%-*CI* = confidence intervals (2.5%, 97.5%); *AIC* = Akaike Information Criterion; *BIC* = Bayesian Information Criterion; *SE* = standard error; *df* = degrees of freedom; *logLik* = log-likelihood; *b* = unstandardized regression coefficient. Significant *p*-values are bold and marked: *: *p* ≤ .050, **: *p* ≤ .010, ***: *p* ≤ .001.

All categorical predictors were effect-coded as follows to aid interpretation of coefficient signs: Age group: reference = 18–30 (1), levels increasing up to >60 (5); Employment status: 0 = part-time, 1 = full-time; Contact with COVID-19: 0 = no, 1 = yes; Single parent: 0 = no, 1 = yes; Living alone: 0 = no, 1 = yes; Children: 0 = no, 1 = yes, but not in same household, 2 = yes, and in the same household; Caregiving for relatives: 0 = no, 1 = yes, but not in same household, 2 = yes, and in the same household. The first category of each categorial factor served as the reference group and is reflected in the intercept.

**model fit and comparison.** The LMM achieved a good fit to the data. The *R² marginal* (.56) suggested that fixed effects explained 56.0% of the variance in WFC, while the *R² conditional* (.92) showed that fixed and random effects together explained 92.0% of the variance.

**model diagnostics.** Residual diagnostics confirmed the adequacy of the model. The residuals followed a normal distribution, as evidenced by both the Kolmogorov-Smirnov test (*D* = .06, *p* = .400) and visual inspection of the residuals’ distribution plotted in a q-q plot (see Figure B). Additionally, the residuals were evenly distributed, indicating no evidence of heteroscedasticity. Multicollinearity was evaluated using Variance Inflation Factor (*VIF*) values, all of which were below 4.31, confirming that multicollinearity was not a concern in the model. This ensured the stability and reliability of parameter estimates. The random-effect structure revealed substantial variability at the participant level (*σ²* = .43, *SD* = .66), while residual variance (*σ²* = .10, *SD* = .31) highlighted within-participant variability over time.

**Figure B.** Q-Q Plot of the residuals from the LMM
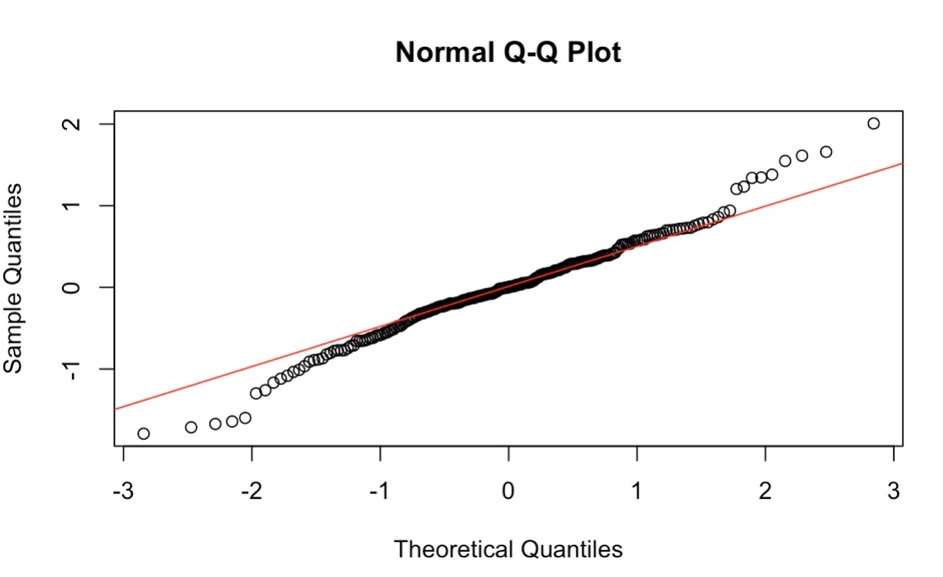


We deliberately retained the full set of theoretically and empirically grounded predictors (drawn from the extant WFC-literature) in our LMM, despite the relatively small number of complete cases. Our focus was on examining these key predictors’ contributions, even if it meant potentially sacrificing some model fit. Our primary aim was to investigate whether the determinants of WFC identified in prior research similarly manifest under the unique conditions of the COVID‐19 pandemic. As such, we refrain from post hoc model reduction via likelihood‐ratio testing and instead emphasize effect‐size estimation and confidence intervals over binary significance thresholds. We acknowledge possibly limited statistical power inherent in our sample; accordingly, we transparently frame our findings as preliminary insights rather than definitive inferences. By situating our approach within a theory‐driven design and by comparing directional patterns across predictors and with analogous studies, we provide a coherent, replicable foundation for future research in pandemic contexts.

Although our longitudinal mixed-effects model included multiple lagged covariates and interaction terms, resulting in a relatively complex specification for *n* = 188 clusters, residual diagnostics did not reveal severe departures from normality or heteroskedasticity. As a supplementary check, we performed a parametric bootstrap with 1,000 replications and observed minimal bias in the fixed-effect estimates and standard errors closely matching the model’s own satterthwaite-based intervals (see Table B). This additional analysis supports the robustness of our inferences but is not critical to the validity of the primary results. This supplementary analysis reinforces our confidence in the model's estimates despite the limited sample size.

**Table B.** Bootstrap statistics and standard errors for fixed effects estimates from the LMM

| parameter | original estimate | bias | bootstrap std. error |
| --- | --- | --- | --- |
| (intercept) | 1.447 | .017 | .442 |
| timepoint | .008 | .002 | .049 |
| workload | .045 | .001 | .019 |
| exhaustion | .057 | .001 | .027 |
| fear | .051 | -.000 | .027 |
| moral concerns | .058 | -.000 | .023 |
| institutional trust | .023 | .000 | .031 |
| caregiving for relatives | .027 | -.005 | .167 |
| caregiving for relatives_lag | .038 | -.000 | .181 |
| contact with COVID-19 | .230 | .005 | .110 |
| contact with COVID-19_lag | .298 | -.001 | .110 |
| age group | -.120 | .002 | .064 |
| workload_lag | .016 | -.001 | .017 |
| exhaustion_lag | .038 | -.001 | .021 |
| fear_lag | .015 | .000 | .020 |
| moral concerns_lag | .002 | -.000 | .022 |
| institutional trust_lag | .043 | -.001 | .026 |
| employment status | -.103 | -.004 | .176 |
| employment status_lag | .367 | -.003 | .188 |
| children | .217 | -.013 | .219 |
| children_lag | .187 | .010 | .215 |
| living alone | .305 | -.006 | .286 |
| living alone_lag | -.275 | .011 | .282 |
| single parent | -.608 | -.019 | .311 |
| single parent_lag | .593 | -.007 | .324 |
| timepoint x workload | .005 | -.000 | .016 |
| timepoint x exhaustion | .016 | -.001 | .022 |
| timepoint x fear | .012 | .001 | .021 |
| timepoint x moral concerns | -.040 | .001 | .024 |
| timepoint x institutional trust | -.067 | -.000 | .025 |

**conclusion.** The model selection process combined theoretical considerations, diagnostic checks, and empirical validation to ensure a robust and interpretable LMM. This analysis provides a comprehensive framework for understanding the factors influencing WFC over time. The choice to center variables, exclude random effects for time due to negligible variance, and incorporate lagged predictors underscores the methodical approach to balancing complexity and interpretability in the modeling process.
